# Exome sequencing identifies novel compound heterozygous mutations in GJB3 gene that cause erythrokeratodermia variabilis et progressiva

**DOI:** 10.1111/ajd.12887

**Published:** 2018-07-10

**Authors:** Yongqiong Deng, Hong Wang, Yunzhu Mou, Qi Zeng, Xia Xiong

**Affiliations:** ^1^ Department of Dermatology & STD The Affiliated Hospital of Southwest Medical University Luzhou Sichuan China; ^2^ Department of Dermatology & STD Affiliated Hospital of North Sichuan Medical College Nanchong Sichuan China; ^3^ Pediatric Department Peking University First Hospital Beijing China

Dear Editor,

Erythrokeratodermia variabilis et progressiva (EKPV) is a group of a rare genodermatosis characterized by the coexistence of migrating red patches resembling a geographic map and localized or generalized hyperkeratosis, which affect the face, buttocks and extensor aspects of the limbs.^1^, ^2^ According to previous studies, EKVP was mainly inherited in an autosomal dominant trait. Mutations associated with EKPV have been identified in *GJB3, GJB4,* and *GJA1* genes encoding connexins (Cx) 31, 30.3, and 43. Here, we reported a novel compound heterozygous mutations of GJB3 gene in a Chinese EKPV family with autosomal recessive inheritance pattern, which was only reported in three families.^3^, ^4^, ^5^

Twelve of 30 individuals in this Chinese EKPV family were investigated. There were three patients and nine unaffected members. Genomic DNA was isolated from peripheral blood using a DNA extraction kit (QIAamp DNA Blood Mini Kit, Qiagen, Hilden, Germany). Three prevalent EKPV associated genes, GJB3, GJB4, and GJA1 were screened for candidate mutations in family members. The candidate mutations were verified in 100 healthy controls.

**Figure 1:**
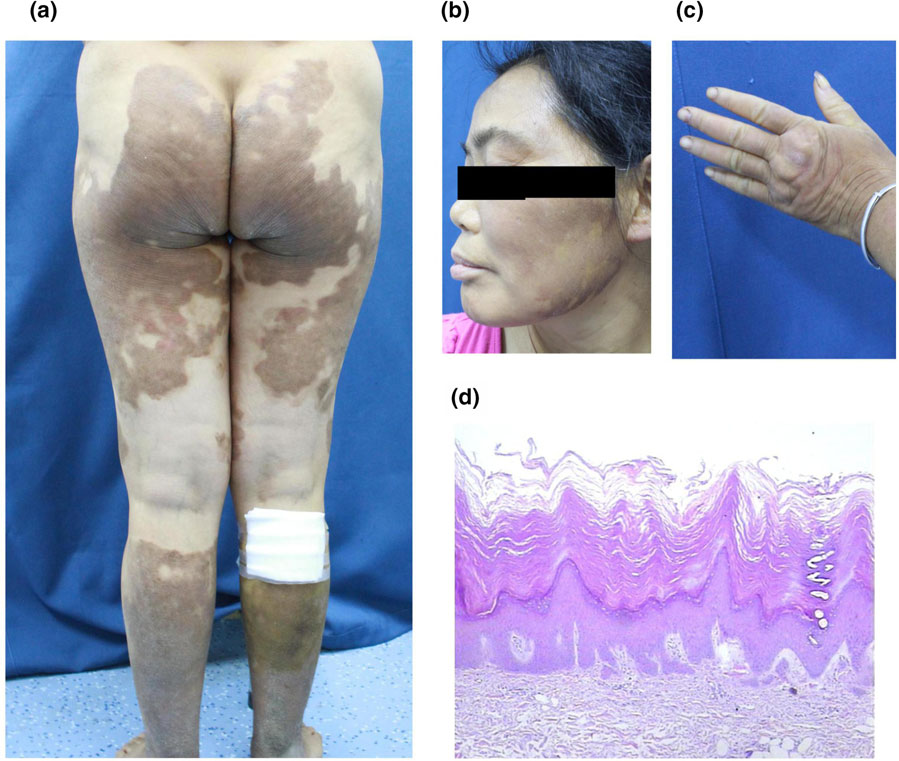
Clinical and histological manifestation of the index patient. (a) Fixed hyperkeratotic plaques on the limbs and buttocks; (b) Brown hyperkeratotic plaques on the face; (c) Lesions on the hand; (d) Ortho‐hyperkeratosis, papillomatosis, and light superficial lyphocyte infiltration in the dermis (HE ×10).

**Figure 2:**
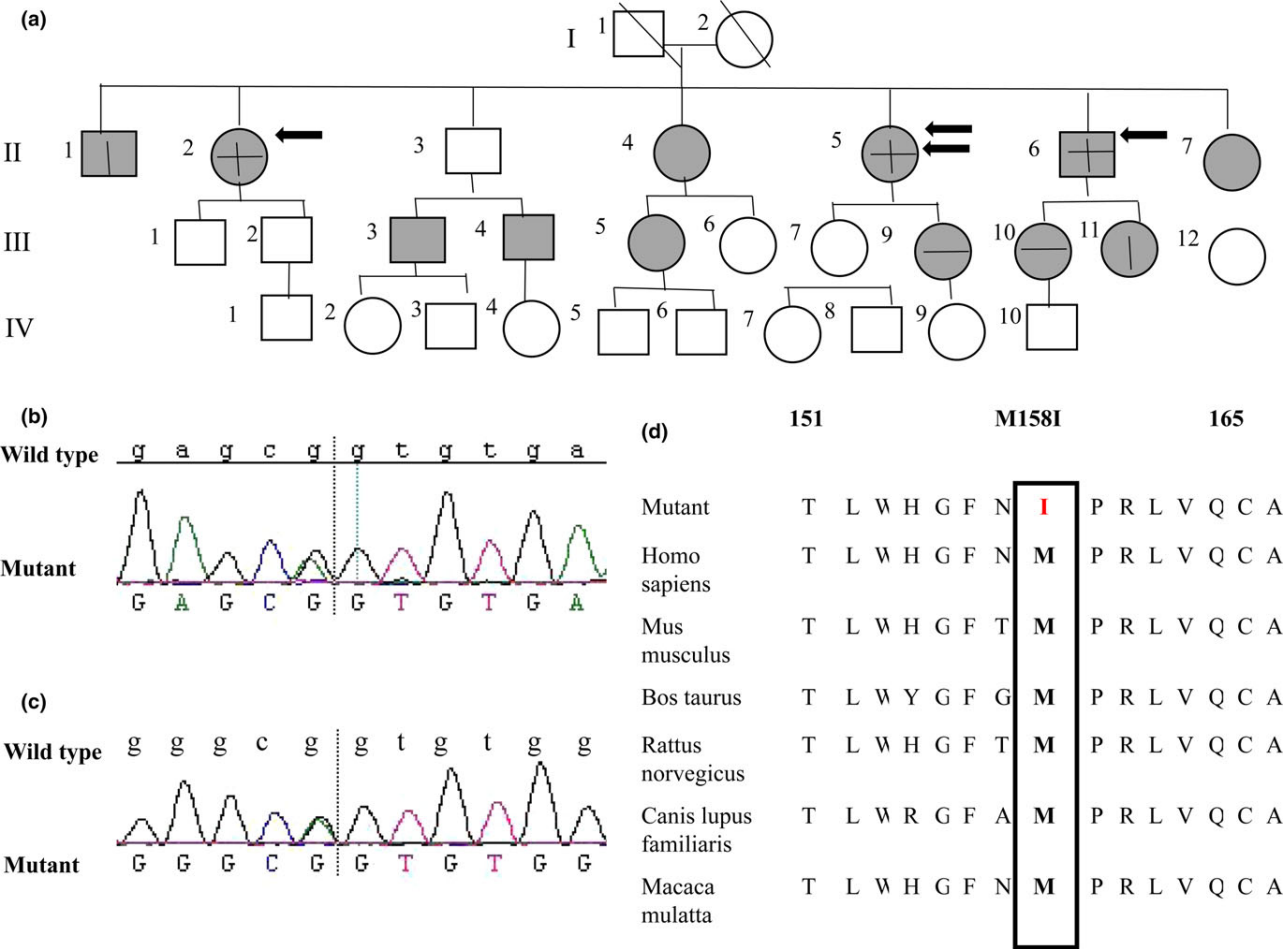
(a) The pedigree chart of the Chinese EKPV family; Circles and box with gray filling represented the tested family members, single‐ and double‐headed arrow indicated patients and the index people, respectively. Horizontal and vertical lines in the box and circle showed the mutations of c. 34 G>A and c. 474 G>A, respectively. (b) A Heterozygous mutation in GJB3 in patients: c. 34 G>A (p.G12S); (c) A Heterozygous mutation in GJB3 in patients: c. 474 G>A (p.M158I); (d) The methionine at position 158 of amino acid chain in Cx31 is highly conserved throughout various species.

The index patient was a 49‐year‐old female with fixed hyperkeratotic plaques on her face, limbs, buttocks, and chest (Fig. 1a–c). Lesions were symmetrically distributed and brownish, characterized by thick dark brown scales and sharp outline. Palmoplantar keratoderma was detected. She had also noticed transient erythematous patches changing in size and sharp and lasting for a few days. Her condition started at the age of 1 year. During the first decade of life, the erythema decreased in intensity, while the diffusion of hyperatotic areas increased. The lesions tended to worsen in winter and improved in summer. The histopathological examination showed ortho‐hyperkeratosis, papillomatosis, and light superficial lymphocyte infiltration in the dermis (Fig. 1d). Two further family members suffered lighter phenotype, compared to the index patient. The other family members were not affected by this disease, nor by any other dermatological conditions. The pedigree chart is illustrated in Fig. 2a. None of the study cases had hearing loss.

The detected genotypes of the family members were shown in Table 1. Mutations analysis showed novel compound heterozygous mutations in *GJB3* gene of the patients as follows: two missense mutations in exon 2, c. 34 G>A (p.G12S), c. 474 G>A (p.M158I) were associated with EKPV in this Chinese family (Fig. 2b,c). The two mutations were derived from homologous chromosomes inherited by patient's parents, respectively. It has been reported that Clycine 12 lies in the cytoplasmic N‐terminal domain in a stretch of six highly conserved residues, and is invariably present at this position in connexins across many species. As for methionine 158, the mutation (red I) also involves a well‐conserved residue (boxed) and is located within the second E2 domain (Fig. 2d).

**Table 1:** The genotype of the family members

Family members	GJB3					GJB4			GJA1	
	34 (G)	866 (G)	1182 (G)	1372 (G)	474 (G)	933 (A)	1140 (A)	1830 (A)	129 (A)	683 (T)
III‐3	G/G	G/G	C/C	C/C	G/G	A/A	A/A	A/C	A/A	T/T
III‐10	A/G	A/G	G/G	G/G	G/G	A/A	A/A	A/C	A/G	T/T
III‐11	G/G	G/G	C/G	C/G	A/G	A/C	A/A	A/C	A/G	T/T
III‐5	G/G	A/G	C/G	C/G	G/G	A/A	A/A	A/C	A/A	T/A
III‐9	A/G	G/G	G/G	G/G	G/G	A/A	A/G	C/C	A/A	T/T
II‐2	A/G	G/G	C/G	C/G	A/G	A/A	A/A	A/C	A/A	T/T
II‐1	G/G	A/G	C/G	C/G	A/G	A/C	A/G	A/C	A/A	T/T
II‐4	G/G	A/G	C/G	C/G	G/G	A/C	A/G	C/C	A/A	T/A
II‐7	G/G	A/G	C/G	C/G	G/G	A/C	A/G	A/C	A/A	T/A
II‐5	A/G	G/G	C/G	C/G	A/G	A/A	A/A	A/C	A/A	T/A
II‐6	A/G	G/G	C/G	C/G	A/G	A/A	A/A	A/C	A/A	T/T
III‐4	G/G	A/G	C/G	C/G	G/G	A/C	A/G	C/C	A/A	T/T

In the family, 2 unaffected members harbored individual heterozygous mutations of c.34 G>A (p.G12S), which has been detected to cause the EKVP phenotype.^6^ The heterogeneous mutation of c.34 G>C has also been reported to be a culprit of EKPV.^7^ In Chinese families, only the combination of heterozygous mutation G34A with G474 has led to the EKPV phenotype. Intra‐ and inter‐familial clinical and genetic heterogeneity has been reported in EKVP, which suggests the effects of modifier genes, epigenetics, and environmental factors. All connexin proteins share a common structure consisting of four trans‐membrane (M1–M4) linked by one cytoplasmic (CL) and two extracellular loops (E1, E2); and N‐ and C‐termini locating on the cytoplasmic side (NT, CT). The E1 domain is important for formation of gap junction channel and the E2 domain is important for docking compatibility in heterotypic channels in connexin family. There are three reports of EKPV cases harboring autosomal dominant mutations in E1 domain.^8^, ^9^, ^10^ G474A in our family is the first pathogenic mutation reported in the E2 domain of Cx31. Mutations in the E2 domain were thought to be associated with autosomal recessive nonsyndromic hearing loss (ADNSHL). In the family, 4 family members harboring G474 showed no hearing loss.

In summary, to our knowledge, we have identified novel compound heterozygous mutations in GJB3 and detected the first pathogenic mutation in E2 domain of Cx31, which was associated with EKPV phenotype.
